# Research Progress in Tunable Fiber Lasers Based on Multimode Interference Filters

**DOI:** 10.3390/mi14112026

**Published:** 2023-10-30

**Authors:** Liqiang Zhang, Kexin Zhu, Yicun Yao, Xiuying Tian, Hailong Xu, Zhaogang Nie

**Affiliations:** 1 School of Physics Science and Information Technology, Liaocheng University, Liaocheng 252000, China; 2220110501@stu.lcu.edu.cn (K.Z.); yaoyicun@lcu.edu.cn (Y.Y.); 2120110521@stu.lcu.edu.cn (X.T.); 2220110605@stu.lcu.edu.cn (H.X.); 2 Key Laboratory of Optical Communication Science and Technology of Shandong Province, Liaocheng University, Liaocheng 252000, China

**Keywords:** tunable fiber laser, multimode interference filter, multimode fiber, no-core fiber, multi-core fiber, tapered fiber

## Abstract

Tunable fiber lasers have the advantages of good beam quality, high integration, and adjustable output wavelength, and they are widely used in fields such as optical fiber communication and optical fiber sensing. The fiber filter is one of the key components of tunable fiber lasers. Among the various filters currently used, multimode interference filters have the advantages of simple structure, convenient implementation, flexible tuning methods, and convenient spectral range design. The structures of multimode interference filters based on multimode fibers, no-core fibers, multi-core fibers, tapered fibers, and other special fibers are introduced in this paper. The working principles and tuning methods are analyzed and the research progress of tunable fiber lasers based on these filters is summarized. Finally, the development trend of tunable fiber lasers based on multimode interference filters is discussed. The rapid development and applications of multimode interference filters can help improve the performance of continuous and pulse lasers as well as promote the practicality of tunable fiber lasers.

## 1. Introduction

Fiber lasers possess the advantages of good beam quality, high conversion efficiency, excellent heat dissipation performance, and good maintainability. They are widely used in fields such as mechanical processing, biomedical treatment, high-capacity communication, as well as defense and military. Tunable fiber lasers, where the output wavelengths are adjustable, are one of the most important branches of fiber lasers. In the field of optical communication, the demand for high-speed communication in emerging industries such as the Internet of Things and smart cities is constantly increasing. The tunable laser is one of the key components for dense wavelength division multiplexing systems and one of the core tools to enhance the flexibility of communication networks. In the field of fiber sensing, the absorption spectra of different gases are located in different wavelengths. The concentration distribution of various gases may be actively detected with tunable lasers. Thus, tunable fiber lasers have important applications in atmospheric pollution monitoring, hazardous gas detection, and flammable gas leakage warning. In addition, tunable lasers are also widely used in areas such as autonomous driving, ultrafast spectroscopy, and microscopy imaging.

The tuning methods of tunable fiber lasers are usually divided into three categories. One is to insert wavelength selection devices such as optical filters into the fiber lasers, and tunable wavelength will be achieved by adjusting the loss of different wavelengths [1]. The second method relies on nonlinear effects such as stimulated Raman scattering [2] or stimulated Brillouin scattering [3], and wavelength conversion or tunability is realized during optical transmission. The third method is to change the transition energy level by changing the length or doping concentration of the gain fiber, thereby changing the working wavelength of the laser [4]. Among these three methods, inserting a filter into the laser system is simple and easy to implement. In particular, fiber filters are of great significance for designing tunable lasers with all fiber structures. So far, fiber gratings [5], birefringent Lyot filters based on polarization-maintaining fibers [6], as well as fiber interferometers [7] have all been used as wavelength selection devices to achieve tunable output wavelengths.

In recent years, with the advancement in fiber drawing and processing technology, multimode interference filters implemented using interference between different modes in fibers have attracted widespread attention [8,9]. The common method to fabricate a multimode interference filter is to fuse a section of fiber with a special structure between two sections of single-mode fibers (SMFs). Different high-order modes are excited at the first fusion joint, which then propagate with different transmission constants in the special fiber. Interference occurs when they are coupled back into the SMF at the second fusion joint. Multimode interference filters have advantages such as simple structure, being easy to integrate with other fiber devices, and convenient spectral range design. This article reviews the research progress of tunable fiber lasers based on multimode interference filters, and the structures and tuning methods of multimode interference filters fabricated with multimode fibers, no-core fibers, multi-core fibers, tapered fibers, and other special fibers are introduced. Finally, the development trend and application prospects of tunable fiber lasers based on interference filters are presented.

## 2. Tunable Fiber Lasers Based on Multimode Fiber Interference Filters

### 2.1. Structure and Working Principle of Interference Filter Based on Multimode Fiber

**Figure 1:**
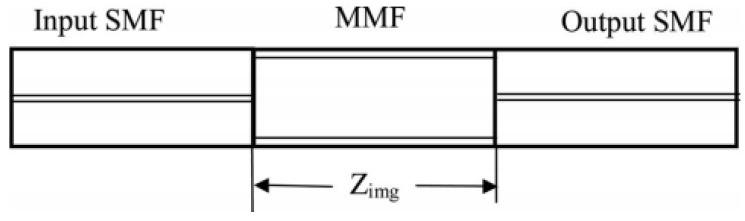
Structure of SMF-MMF-SMF filter [8].

An interference filter based on multimode fiber is usually fabricated by fusing a segment of graded-index multimode fiber (GIMF) or step-index multimode fiber (SIMF) between two SMFs, forming a single-mode–fiber-multimode–fiber-single-mode fiber (SMF-MMF-SMF) structure, as shown in Figure 1 [8]. The working principle of this filter is explained as follows. The light transmitted along the input SMF enters the multimode fiber with an approximately Gaussian-shaped field distribution, and multiple high-order guided modes are excited at the first fusion point. These modes propagate in the multimode fiber with different transmission constants, resulting in interference with each other due to the accumulated phase difference. When the phase shift of all modes is an integer multiple of 2π, the interference between different modes forms the self-imaging of input light [9,10]. Thus, the length of the MMF has to be precisely cleaved to have a self-image right at the facet of the output SMF. Usually, the length of the MMF can be calculated using [9]

(1) $$z=m\left(\frac{3L_{\pi }}{4}\right),\;m=0,1,2\cdots$$

where m is the order of self-imaging. $L_{\pi }$ is the beat length, which is given by

(2) $$L_{\pi }≅\frac{4n_{core}a^{2}}{3\lambda_{0}}.$$

In Equation (2), $n_{core}$ and a correspond to the effective refractive index and the diameter of the MMF core. When the input light is broadband light, the oscillating interference curve will be measured in the output SMF. From Equations (1) and (2), the peak wavelengths of the transmission curve are determined by the following equation [9]:

(3) $$\lambda_{0}=m\frac{n_{core}a^{2}}{z},\;m=0,1,2\cdots$$

According to Equation (3), the peak transmission wavelength of the filter is related to the length, effective refractive index, and radius of the multimode fiber. S. M. Tripathi et al. [11] discussed the dependence of filter bandwidth on stretching and temperature. The results show that the filter could be switched between band pass and band stop modes, and that the filter bandwidth in each mode could be dynamically tuned. T. Walbaum et al. [12] analyzed the effects of bending on the transmission spectrum and polarization state both theoretically and experimentally, and a filter with a continuous tuning range of 13.6 nm and an 86% peak transmission was realized.

### 2.2. Tuning Method of Interference Filter Based on Multimode Fiber

For SMF-MMF-SMF filters, the commonly used tuning methods are stretching [10,13,14], bending [15,16,17,18,19,20,21,22], or wrapping into a polarization controller (PC) [23,24,25]. Both mode-locked and continuous wave (CW) fiber lasers have been reported based on MMF filters. Table 1 gives a summary of the literature reports on tunable fiber lasers based on SMF-MMF-SMF filters. The tuning ranges are the results of single wavelengths. Dual- or triple-wavelength tuning results are not included.

#### 2.2.1. Stretching

**Figure 2:**
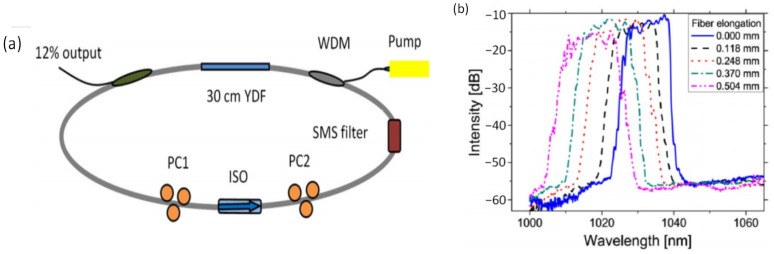
(**a**) Tunable Yb-doped fiber laser based on SMF-MMF-SMF filter; (**b**) Tunable results of the dissipative soliton fiber laser [10].

Stretching changes the length of the MMF, and the stress caused by stretching changes the effective refractive index of the transmission modes propagating in the multimode fiber, thereby changing the peak transmission wavelengths of the filter. In 2012, L. Zhang et al. [10] demonstrated a dissipative soliton fiber laser using the SMF-MMF-SMF filter as the wavelength selection device, as shown in Figure 2. By applying a tensile strain to the filter, the laser wavelength was tunable within a 12 nm range. In 2019, H. Li et al. [13] constructed a Tm-doped mode-locked fiber laser using the SMF-MMF-SMF structure as a saturable absorber and wavelength selection device. Continuously tunable mode-locked pulses were experimentally achieved by varying the stretching length of the multimode fiber. In 2020, J. Yu et al. [14] designed an optical parametric oscillator based on two cascaded SMF-MMF-SMF filters. By stretching the MMF in the SMF-MMF-SMF device, the oscillator wavelength was tunable in the range from 1642.5 nm to 1655.4 nm.

#### 2.2.2. Bending

**Figure 3:**
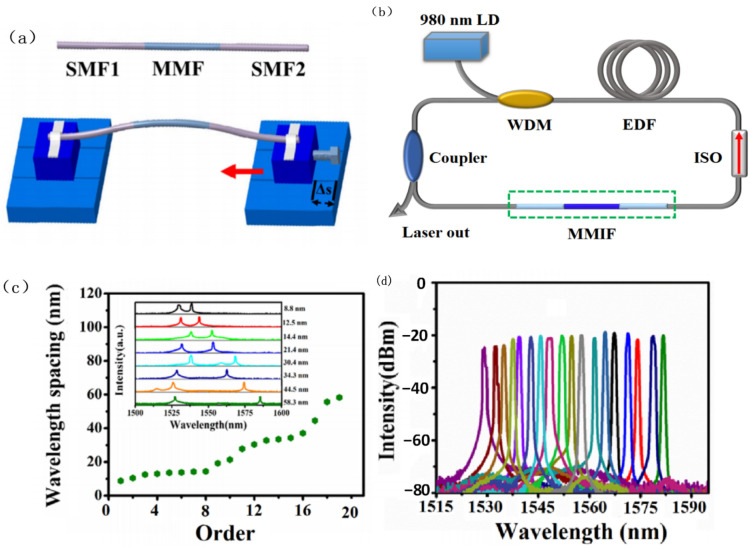
(**a**) Tuning principle of SMF-MMF-SMF filter by bending; (**b**) Experimental setup diagram of the tunable fiber laser; (**c**) Output spectra of the interval-adjustable dual-wavelength fiber laser; (**d**) Output spectra of the tunable single-wavelength fiber laser [20].

Similar to stretching, bending changes the propagation constants of each mode propagating in the MMF, thereby changing the transmission wavelengths of the filter. T. Walbaum et al. [18] reported a nonlinear polarization–rotation mode-locked erbium-doped fiber laser with an SMF-MMF-SMF filter, and the spectra were tunable in a range of 11.6 nm by bending the fiber filter. In this report, a Sigma cavity was used to avoid the impact of polarization state changes introduced by bending. N. Li et al. [20] reported an erbium-doped fiber laser based on an SMF-MMF-SMF filter in 2018. When the laser operated in the single wavelength state, the wavelength could be tuned with a range of 52 nm, and when the laser operated in a dual wavelength state, wavelength spacing could be tuned from 9 to 58 nm by bending the filter. Figure 3 shows the structure diagram of the constructed laser and the tuning results.

In addition to the fiber communication band, 1 μm band [22] and 2 μm band [16,17] tunable fiber lasers have also been reported based on these filters. In ref. [22], a wavelength-tunable passively mode-locked Yb-doped fiber laser was demonstrated. The central wavelength could be tuned by moving the horizontally adjustable platforms to bend the SMF-GIMF-SMF filter, and the tuning range was from 1031.99 nm to 1039.32 nm. In ref. [16], H. Li reported an all-fiber Tm fiber laser, where the SMF-SIMF-GIMF-SMF structure was used as both the saturable absorber and filter. By varying the curvature of the SMF-SIMF-GIMF-SMF structure, the wavelength of the solitons could be continuously varied in the range of 1835–1886 nm.

#### 2.2.3. Wrapping into a Polarization Controller

Tunable fiber lasers have also been realized by wrapping the SMF-MMF-SMF filter into a three-paddles polarization controller.

**Figure 4:**
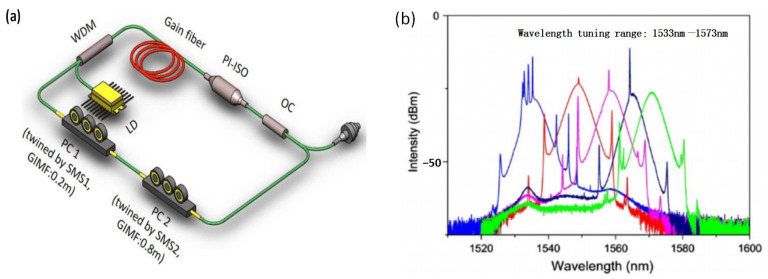
(**a**) Experimental setup diagram of the tunable mode-locked fiber laser; (**b**) Output spectra of the tunable mode-locked fiber laser [24].

In the SMF-MMF-SMF filter, high-order modes are excited when the input light enters the MMF from the SMF, and different high-order modes accumulate different phase shifts when they are propagating in the multimode fiber, resulting in interference phenomena when they are coupled back into the second SMF. When the SMF-MMF-SMF structure is wrapped into a polarization controller, the fiber undergoes stretching and bending due to twisting, resulting in the change in the propagation constants of each mode propagating in the MMF. Changing the angle of the polarization controller paddles will change the degree of stretching and bending, thereby affecting the phase difference between different modes and causing changes in the transmission peaks of the interference curve. In 2019, H. Zhang et al. [24] reported a wavelength tunable passively mode-locked fiber laser based on two cascaded SMF-MMF-SMF structures, as shown in Figure 4a. Each of the SMF-MMF-SMF structures was wrapped into a three-paddles polarization controller, and a tuning range from 1533 nm to 1573 nm was achieved by mechanically tuning the orientation angles of the paddles of the polarization controllers (Figure 4b). In 2022, Y. Qi et al. [25] adopted a wrapped SMF-MMF-SMF structure as both saturable absorber and comb filter in a mode-locked fiber laser, and continuously tunable multi-wavelength mode-locked pulses were obtained by varying the paddles’ orientation.

**Figure 5:**
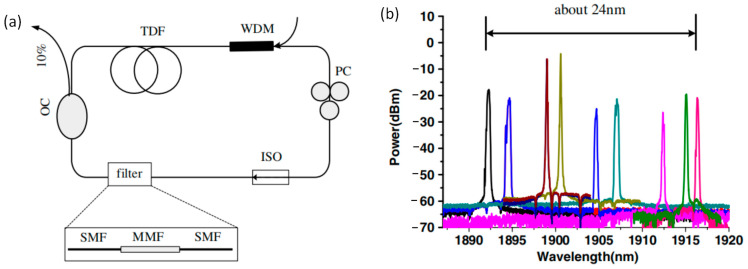
(**a**) Experimental setup diagram of the Tm-doped fiber laser; (**b**) Measured spectra of the single-wavelength laser by adjusting the PC and rotating the MMF [26].

In addition, the fiber laser cavity artificial birefringence filter will also affect the oscillating wavelengths. By adjusting the PC inserted into the fiber laser, the transmission spectrum of the SMF-MMF-SMF is modulated, so the oscillating wavelength can also be tuned or switched by adjusting the PC in the cavity [26,27,28]. In ref. [26], a multiwavelength Tm-doped fiber laser was proposed and experimentally demonstrated. The wavelength could be tuned by adjusting the PC and rotating the MMF in the SMF-MMF-SMF structure. The experimental setup diagram and wavelength-tuning results are shown in Figure 5. Figure 5a is the ring cavity configuration of the Tm-doped fiber laser, and Figure 5b is the measured spectra of the single-wavelength laser by adjusting the PC and rotating the MMF. The wavelength could be changed from 1892.66 nm to 1916.04 nm, with a tuning range of 24 nm.

In mode-locked fiber lasers, the wavelengths could be switched by adjusting the PC in the cavity. In ref. [27], J. Chen et al. reported the results of wavelength-switchable bound solitons in a passively mode-locked fiber laser based on tapered GIMF. By adjusting the PC inserted in the cavity, the central wavelength of the fiber laser could be switched between 1565 nm and 1595 nm. In ref. [28], H. Li et al. demonstrated an all-fiber multiwavelength mode-locked thulium-doped fiber employing a cascaded SMF-SIMF-GIMF-SMF structure, acting both as a filter and as a saturable absorber. Stable tri-wavelength (1857/1897/1934 nm) mode-locking operation was obtained. The tri-wavelength mode-locking state could be switched to single- or dual-wavelength mode-locking state by rotating the PC properly. Changing the ambient temperature [11] has also been used to achieve tunable wavelengths.

## 3. Tunable Fiber Lasers Based on No-Core Fiber Interference Filters

### 3.1. Structure and Working Principle of No-Core Fiber Interference Filter

**Figure 6:**
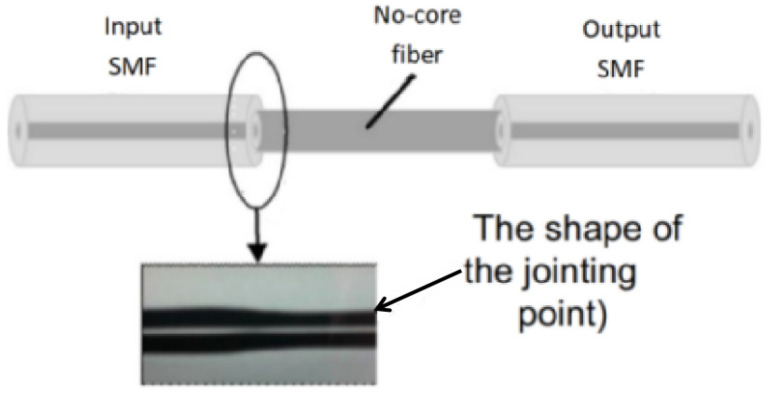
Structure of the SMF-NCF-SMF filter [29].

With the development of fiber preparation technology, various special fibers with different structures have been used to prepare multimode interference filters. A commonly used type of fiber is the no-core fiber (NCF). As shown in Figure 6, a single-mode fiber no-core-fiber single-mode-fiber (SMF-NCF-SMF) interference filter is formed by fusing a section of NCF between two SMFs. The working principle of this kind interference filter is similar to the SMF-MMF-SMF filter. When incident light enters the NCF, higher-order modes are excited, and different modes undergo different optical paths during propagating in the NCF. These higher-order modes cause interference between each other when they are coupled back into the following SMF [29].

### 3.2. Tuning Method of No-Core Fiber Interference Filter

**Figure 7:**
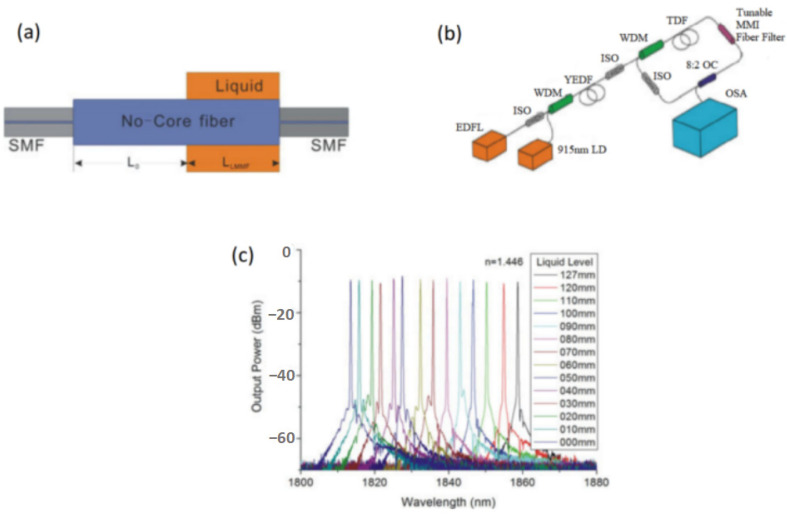
(**a**) Structure of the tunable SMF-NCF-SMF filter; (**b**) Experimental setup diagram of the tunable Tm-doped fiber laser; (**c**) Tuning results of the Tm-doped fiber laser [31].

When the refractive index of the surrounding environment of the NCF changes, the transmission constant of the light propagating in the fiber changes. Therefore, an advantage of the multimode interference filters based on NCF is that wavelength tuning can be easily achieved by changing the environmental refractive index [29,30,31]. In 2014, L. Ma et al. [29] demonstrated a tunable erbium-doped all-fiber laser, where a thinner NCF with a diameter of 104 μm was used to fabricate the SMF-NCF-SMF filter. The filter was sensitive to the change in the environmental refractive index. In the experiment, the filter was covered with glycerol solution. When the concentration of glycerol solution was changed from 0% to 78%, the effective refractive index ranged from 1.333 to 1.440, and the oscillating wavelength of the fiber laser changed from 1532 nm to 1564 nm, with a tuning range of 32 nm. In ref. [31], X. Ma et al. fabricated an SMF-NCF-SMF filter, and when the NCF was gradually vertically covered by refractive-index-matching liquid, the peak wavelengths of the transmission curve of the fiber filter were tuned. Using the SMF-NCF-SMF filter, a thulium-doped fiber laser was demonstrated, and the wavelength was tunable in the range from 1831.52 nm to 1858.70 nm. Figure 7 shows the filter structure, experimental setup diagram, and tuning results.

**Figure 8:**
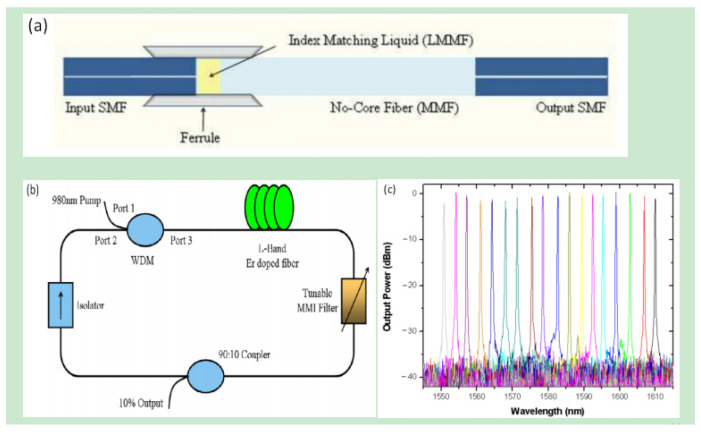
(**a**) Structure of the SMF-NCF-SMF filter based on index-matching liquid; (**b**) Experimental setup diagram of the tunable erbium-doped fiber laser; (**c**) Tuning results [32].

Combined with the refractive-index-matching fluid, the SMF-NCF-SMF filter could also be tuned by changing the length of the liquid. A. Castillo-Guzman et al. [32] reported a widely tunable erbium-doped fiber laser based on the multimode interference effect. The tuning mechanism was based on a fused silica ferrule filled with a refractive-index-matching fluid. The ends of the SMF and NCF were inserted into the ferrule, and their separation was changed to tune the peak wavelength. Figure 8 gives the schematic of the filter, the experimental setup of the tunable erbium-doped fiber laser, and the tuning results.

## 4. Tunable Fiber Lasers Based on Multi-Core Fiber Interference Filters

### 4.1. Structure and Working Principle of Multi-Core Fiber Interference Filter

Different from conventional fiber, there is more than one core in multi-core fibers. According to the number of cores, multi-core fibers can be divided into two-core fibers, four-core fibers, seven-core fibers, and so on. If the distances between the cores are long, each core serves as an independent waveguide, and light propagates independently within these cores. This type of fiber is called a weakly coupling multi-core fiber. If the distances between the cores are close, the coupling between different cores will occur when light is coupled into one core, and this type of fiber is usually called a strong coupling multi-core fiber.

#### 4.1.1. Structure and Working Principle of Strong Coupling Multicore Fiber Interference Filter

**Figure 9:**
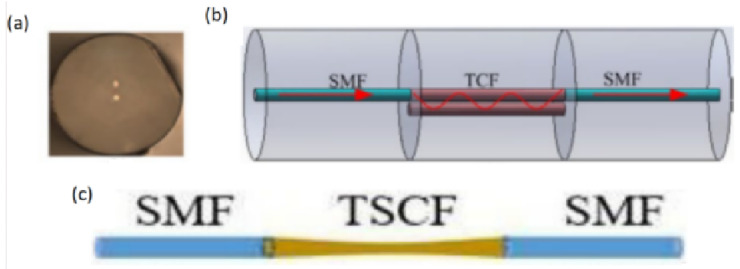
(**a**) Cross-section of the two-core fiber [33]; (**b**) The structure of the interference filter based on two-core fiber [33]; (**c**) Structure of the interference filter based on tapered seven-core fiber [35].

Currently, most tunable lasers based on multi-core fibers are fabricated with strong coupling fibers. Figure 9a shows a cross-section of a two-core fiber (TCF). The TCF filter (Figure 9b) is prepared by fusing a section of TCF between two sections of SMFs [33]. At the first fusion point, the light transmitted in the SMF is coupled with one core of the TCF. Due to the small distance between these two cores, the light is coupled and transmitted in these two cores, and it returns to the SMF at the second fusion point [34]. When a segment of weakly coupled multi-core fiber is heated and tapered, the distance between the cores becomes shorter and shorter, and a strong coupling fiber will be formed. Figure 9c shows a filter prepared with a segment of seven-core fiber, which is made by fusing a tapered seven-core fiber (TSCF) between two SMFs [35]. Taking the TSCF as an example, the working principle of a strong coupling multi-core fiber filter is analyzed as follows.

When light is injected from the middle core of the seven-core fiber, the intensity of the light in the middle core and the six side cores can be expressed as [35]

(4) $${\left|A_{1}\left(z\right)\right|}^{2}=\frac{1}{7}+\frac{6}{7}\cos^{2}\left(\sqrt{7}Cz\right)$$

$${\left|A_{p}\left(z\right)\right|}^{2}=\frac{1}{7}\sin^{2}\left(\sqrt{7}Cz\right)\;p\neq 1$$

in which $n_{1}$ and $n_{2}$ are the refractive indices of the middle-core and side-core modes, while a and d are the core diameter and the spacing between two cores, respectively. $K_{0}$ and $K_{1}$ denote the Henkel functions of order 0 and 1. U, V, and W are represented as U=a2πn1λ2−β2, $V=\frac{2\pi a}{\lambda }\sqrt{n_{1}^{2}-n_{2}^{2}}$, and W=aβ2−2πn2λ2. They are normalized as radial phase constant, normalized radial attenuation constant, and normalized frequency.

**Figure 10:**
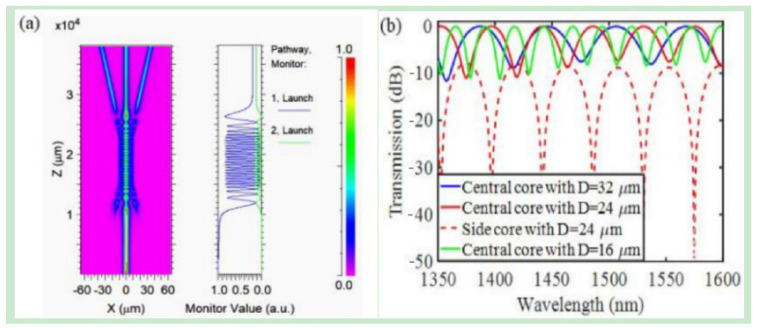
(**a**) The field distribution and normalized power in a tapered seven-core fiber simulated by Rsoft software; (**b**) Transmission spectra of tapered seven-core fiber simulated by Rsoft software [35].

From Equation (4), it can be seen that the middle-core mode and side-core mode oscillate periodically with a phase of π2. Figure 10 shows the normalized light intensity distribution of the strong coupling part and the weak coupling part of the seven-core fiber simulated by Rsoft software (202103). In the strong coupling part of the tapered region, light oscillates in the middle core and side cores are observed, while in the non-tapered part after the tapered region light propagates independently in each core. Figure 10b shows the oscillation spectra in the middle core and the side cores, with a phase difference of  π2, which is consistent with the theoretical analysis results.

Up to now, except for the tapered seven-core fiber, strong coupling multi-core fibers such as three-core fiber [36], two-core erbium-doped gain fiber [37], and two-core photonic crystal fiber [38] have all been used to prepare filters and have achieved tunable output wavelengths in fiber lasers.

#### 4.1.2. Structure and Working Principle of Weakly Coupling Multi-Core Fiber Interference Filter

**Figure 11:**
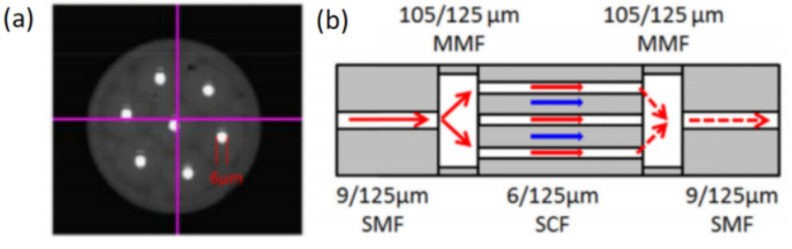
(**a**) Cross-section of the seven-core fiber; (**b**) Working principle of the interference filter based on seven-core fiber [39].

Weakly coupling seven-core fiber has also been used to fabricate interference filters [39], and the structure of the seven-core fiber is shown in Figure 11a. The working principle of the filter is shown in Figure 11b. The light transmitted in the SMF is divided into two parts by the first segment of the MMF, where one part propagates in the core of the seven-core fiber, and the other part propagates in the cladding of the seven-core fiber. These two parts of the light are recoupled back to the SMF after passing through the second segment of the MMF, and interference occurs due to the different effective refractive indices of the light transmitted in the core and cladding.

### 4.2. The Tuning Method of Multi-Core Fiber Interference Filter

**Figure 12:**
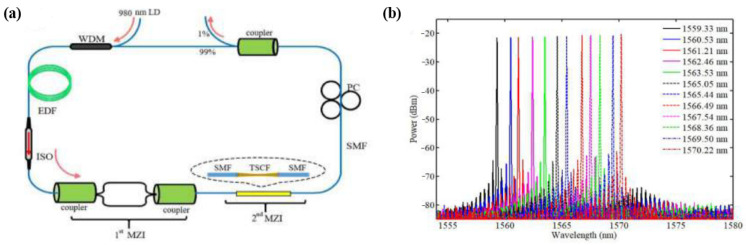
(**a**) Experimental setup diagram of the tunable erbium-doped fiber laser based on tapered seven-core fiber filter; (**b**) Spectra of the tunable fiber laser [35].

The commonly used methods to tune multi-core fiber interference filters are stretching and bending. In 2011, G. Lin et al. [40] proposed a filter based on TCF, where three TCF filters were cascaded to optimize the transmission spectrum of the filter. By applying tension to the filter, the central wavelength of the filter could be adjusted in the range from 1540.8 nm to 1557.8 nm. In 2020, Y. Lv et al. [36] reported an erbium-doped fiber laser based on a three-core photonic crystal fiber (TCPCF), and single-wavelength, dual-wavelength, and three-wavelength tunability were achieved by applying tension, with tuning ranges of 19.58 nm, 10.34 nm, and 6.84 nm, respectively. In 2020, we proposed a narrow-linewidth, wavelength-tunable erbium-doped fiber laser by cascading a Mach–Zehnder interferometer and a TSCF filter [35]. By applying strain to the TSCF, the wavelength could be tuned from 1570.22 nm to 1559.33 nm. Figure 12a,b show the experimental setup and tuning results of the laser, respectively. In 2013, G. Yin et al. [34] reported a fiber laser based on a TCF filter. By bending the filter, the output wavelength of the laser was tunable, with a tuning range of 24 nm. In 2014, a tunable erbium-doped fiber laser was reported based on two cascaded TCF filters. The number of the wavelength could be adjusted by bending one filter, and the central wavelength could be tuned by stretching the other filter [33]. Fiber lasers based on multi-core fibers are summarized in Table 2. The tuning ranges are the results of single wavelengths. Dual- or triple-wavelength tuning results are not included.

## 5. Tunable Fiber Lasers Based on Tapered Fiber Filters

Tapered fibers are widely used in fiber couplers, fiber sensors, and other nonlinear fiber devices. The common method to prepare a tapered fiber is to heat and stretch a segment of traditional fiber. During the stretching process, the diameter of the fiber decreases gradually. According to the structure of the tapered fiber, the interference filters based on tapered fibers can be divided into two types, which we refer to as single-cone filters and double-cone filters.

### 5.1. Structure and Tuning Principle of Single-Cone Interference Filter

There is only one tapered region in the single-cone interference filter. The tapered region supports two or more high-order modes, which couple with each other and are coupled back into the fiber core in the non-tapered region. Interference is generated due to the different transmission constants between different modes. In 2017, S. Celachi et al. [42] calculated the dependence of effective index of excited HE_11_ and HE_12_ modes on the external radius of the tapered region. According to the mode-coupling theory, the normalized output power $P_{out}$ from the HE_11_ mode is described by the follow equation [42]:

(5) $$P_{out}\left(\lambda_{0}\right)=\psi \cos{\left(\phi \right)}^{2}+\zeta \sin{\left(\phi \right)}^{2},$$

where $\Psi =0.999^{2}$ and $\zeta =0.013^{2}$ are the square of the overlap integral between the HE_11_ and HE_12_ supermodes (from the coaxial waveguide) in-quadrature and phase, and the HE_11_ mode of the rod waveguide, respectively. ϕ denotes the accumulated phase difference between these modes after propagating a distance L:

(6) $$\phi =\frac{2\pi }{\lambda_{0}}\int_{0}^{L}\Delta n_{eff}\left(\lambda_{0},z\right)dz.$$

**Figure 13:**
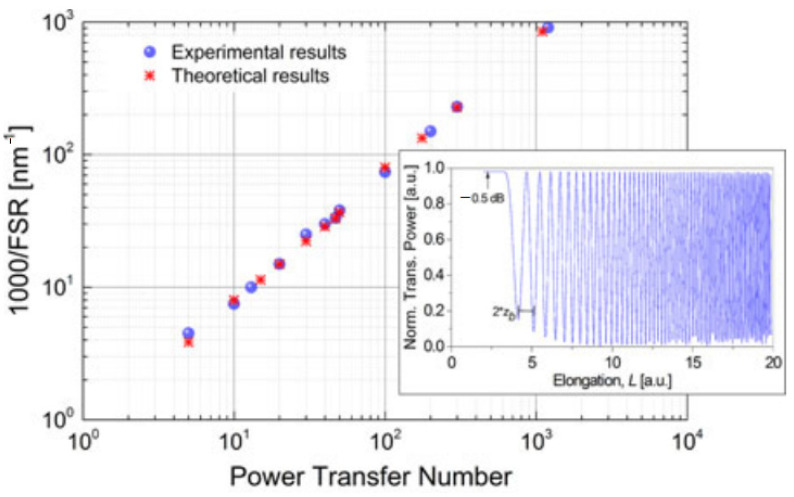
Power transfer number versus FSR measured (blue circles) and simulated (red stars) at λ_0_ = 1.55 μm for tapers assuming different elongations. Inset shows an experimental example of normalized transfer power versus taper elongation [42].

Figure 13 shows the numerical results calculated using Equations (5) and (6), which agree with the experimental results well.

**Figure 14:**
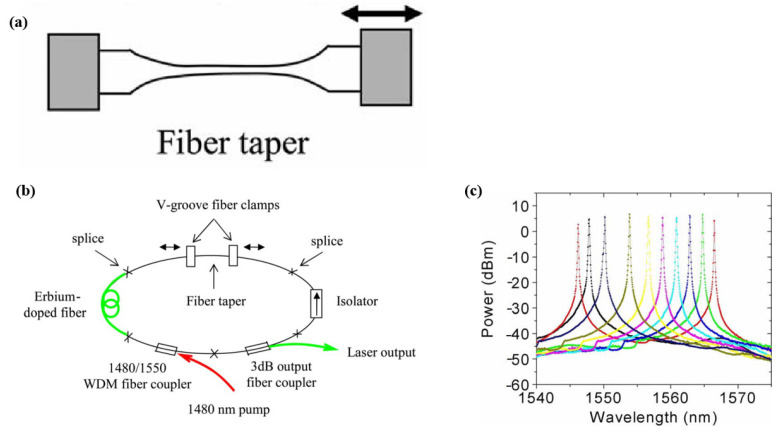
(**a**) Schematic diagram of the tapered fiber filter; (**b**) Tunable fiber laser based on tapered fiber filter; (**c**) Tuning results of the fiber laser [44].

The commonly used method to tune a fiber laser based on single-cone fiber filters is to apply tension to the tapered region [43,44,45,46,47]. In 2006, K. Kieu et al. [44] used this filter as a wavelength-selection device to build an erbium-doped fiber laser. When the stretching reached 180 μm, the output wavelength of the laser was tunable in the range from 1546 nm to 1567 nm. Figure 14 shows the structure diagram of the tapered fiber filter, experimental setup diagram, and tuning results. In 2010, the same team designed a 2 μm band mode-locked fiber laser, where the central wavelength could be tuned by applying tension to the tapered region [43]. In 2017, H. Ahmad et al. [47] constructed a mode-locked fiber laser based on a single-cone interference filter, and when the stretching range of the filter reached 100 μm, the central wavelength of the mode-locked pulse was tunable in the range from 1560 nm to 1556.2 nm. The article also pointed out that, the smaller the diameter and the longer the length of the tapered area, the more sensitive it was to stretching. A summary of the fiber lasers based on single-cone interference filter is shown in Table 3. The tuning ranges are the results of single wavelengths. Dual- or triple-wavelength tuning results are not included.

### 5.2. Structure and Tuning Principle of Double-Cone Interference Filter

**Figure 15:**
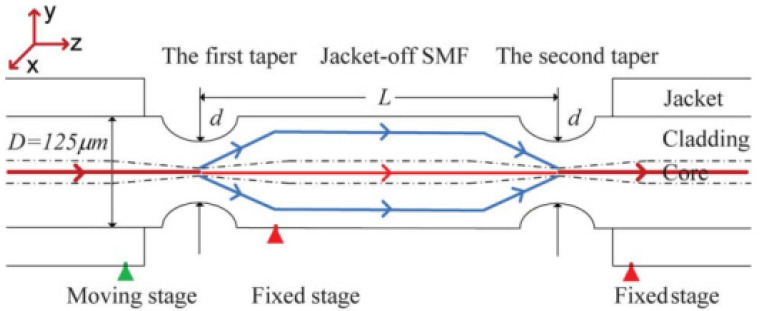
Schematic diagram of the double-cone MZI filter [50].

In a single-cone filter, the transition in the tapered region is generally relatively slow. If it is a steeply changing cone, the angle of the transition region is relatively large, and part of the light will be coupled from the fiber core to the cladding. If another identical steeply changing cone is added a few centimeters after the first one, the light coupled to the cladding will be recoupled back into the fiber core. Due to the different refractive indices of the core mode and cladding mode, interference occurs when the light is coupled back to the core [50]. As shown in Figure 15, this kind of interference filter has two tapered regions. In the first tapered region, part of the light is coupled into fiber cladding, and in the second tapered region, the light is coupled back to the fiber core. The working principle of the double-cone interference filter is similar to that of a Mach–Zender interferometer (MZI), so it is commonly referred to as an MZI filter.

The intensity at the end of the interferometer structure can be expressed as follows [51]:

(7) $$I\approx I_{core}+I_{cladding}=2\sqrt{I_{core}I_{cladding}}\cos\Delta \phi ,$$

where $I_{core}$ denotes the intensity of the core mode, and $I_{cladding}$ is the cladding mode intensity propagating between the first and second tapers. Δϕ denotes the phase difference between core and cladding modes, which is given as follows:

(8) $$\Delta \phi \approx \frac{2\pi \left(n_{core}^{eff}-n_{cladding}^{eff}\right)L}{\lambda }.$$

In Equation (8), $n_{core}^{eff}$ and $n_{cladding}^{eff}$ are the effective refractive indices of the core and cladding modes, respectively. λ is the operation wavelength and L denotes the physical length between these two tapers. The free spectral range of the interferometer is expressed as follows:

(9) $$\Delta \lambda \approx \lambda_{m-1}-\lambda_{m}=\frac{\lambda_{m-1}\lambda_{m}^{}}{\Delta n_{eff}L},$$

where $\lambda_{m-1}$ and $\lambda_{m}$ are the wavelengths of *m*th and (*m*−1)th-order interference dips, respectively. $\Delta n_{eff}$ gives the effective refractive index difference of the core and cladding modes.

**Figure 16:**
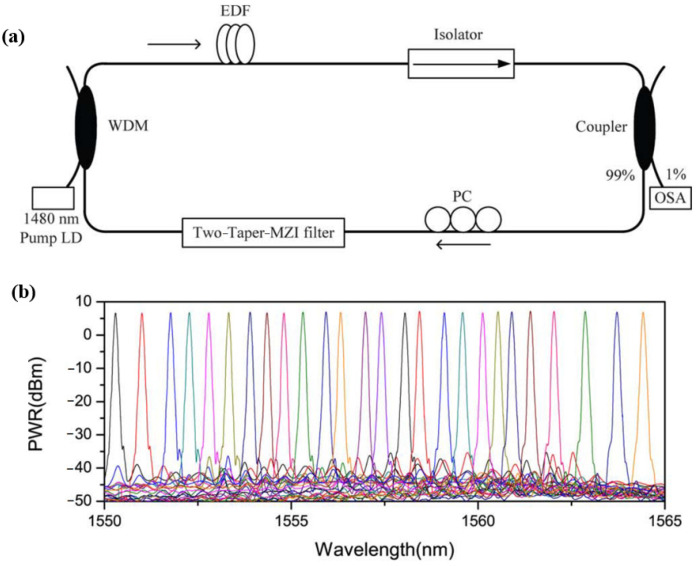
(**a**) Experimental setup diagram of the tunable fiber laser based on a double-cone MZI filter; (**b**) Tuning results after annealing [50].

For double-cone interference filters, the commonly used tuning method is bending [50,51,52,53,54]. When the filter is bent, more high-order modes are excited in the tapered region, thereby changing the interference effect between different modes [50]. In 2010, X. Wang et al. [50] reported a tunable C-band and L-band fiber lasers based on biconical MZI filters, and the tuning range covered the entire C and L-band. Figure 16a,b show the experimental setup of the tunable fiber laser and the tuning results after annealing, respectively. In 2014, M I. Md Ali et al. [53] designed a dual-wavelength fiber laser based on a biconical MZI filter and analyzed the effects of tapered region length on the free spectral range, extinction ratio, and bandwidth of the interference curve. Tunable output wavelengths were obtained by bending one of the tapered regions of the MZI filter. In 2022, G. Salceda-Delgado et al. [51] reported an erbium-doped fiber laser with a double-cone MZI as the wavelength selector, and the central wavelength was tunable from 1527 nm to 1563 nm. Additionally, the laser could emit simultaneous laser line emission, starting from one to four. The stability of the laser was also discussed. 

In addition to bending, changing the refractive index of the liquid surrounding the tapered region or applying tension will also change the characteristics of the filter. In 2014, R. Selva-Aguilar [55] immersed a biconical interferometer in glycerol solution and changed the refractive index of the solution through heating to achieve tunable laser output, and the tuning range was 12 nm. In 2016, H. Ahmad et al. [56] reported a dual-wavelength Yb-doped fiber laser with adjustable wavelength spacing based on a double-cone interferometer. When the stretching amounts were 2 μm, 12 μm, 87 μm, and 190 μm, the dual wavelength intervals were 7.88 nm, 7.62 nm, 11.59 nm, and 7.12 nm, respectively. Fiber lasers based on tapered fiber filters are summarized in Table 3.

## 6. Tunable Fiber Lasers Based on Other Interference Filters

Except for the interference filters mentioned above, there are also filters fabricated with other special fibers or structures, such as four-leaf clover suspended core fibers [59], two cascaded up-taper joints structure [60], photonic crystal fibers [61], and core-offset structure [62].

**Figure 17:**
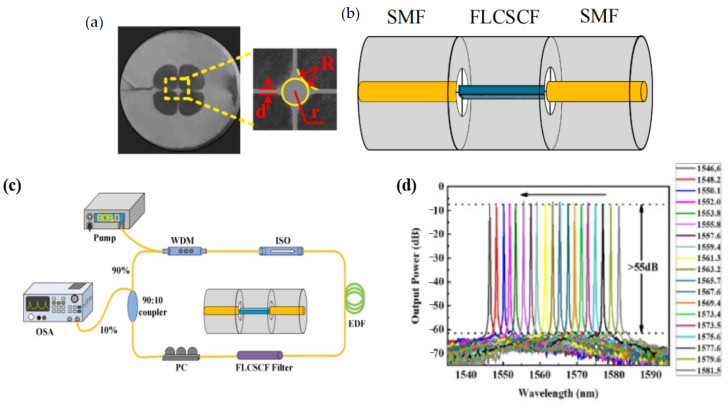
(**a**) The cross-section of the FLCSCF; (**b**) Schematic diagram of the FLCSCF filter; (**c**) Experimental setup of the fiber laser based on FLCSCF filter; (**d**) Tunable single-wavelength lasing output [59].

In 2011, Z. Tang et al. [59] reported a high-performance fiber laser by using a four-leaf clover suspended core fiber (FLCSCF) filter. The filter was fabricated by splicing a segment of FLCSCF between two segments of SMFs, as shown in Figure 17a,b. The working principle of this filter is similar to the SMF-MMF-SMF filter or SMF-NCF-SMF filter. The mode transmitted in the first SMF is diffracted at the fusion point between the SMF and FLCSCF, where high-order core modes and multiple cladding modes are excited in the following FLCSCF. The propagation constants of these core modes and cladding modes are different; thus, a certain phase difference is accumulated after transmitting a distance in the FLCSCF. These modes will converge and recombine with each other at the second fusion point. Inserting the FLCSCF filter in a ring fiber laser cavity, the central wavelength could be tuned from 1581.5 nm to 1546.6 nm by applying strain to the filter. Figure 17c,d are the experimental setup of the fiber laser and the tunable single-wavelength output, respectively.

**Figure 18:**
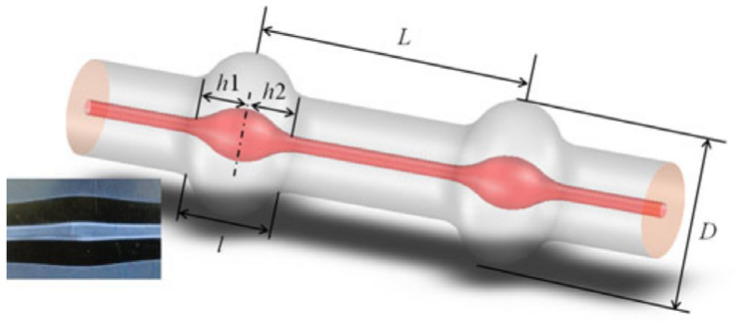
Diagram of two cascaded up-taper joints structure [60].

Multimode interference filters have also been prepared by cascading two up-taper joints (Figure 18) [60]. The up-taper was fabricated by increasing the forward distance infusion splicing using a fusion splicer. The working principle of this filter is similar to that of the tapered dual-cone filter. Cladding modes are excited in the first up-taper point, which will propagate in the cladding of the fiber between these two tapers. The couple of cladding mode back to core mode will happen in the second up-taper point, and the core mode and cladding mode meet and create interference with each other. Fiber lasers based on this filter possess the sensing characteristics that the interferometer possesses. The temperature and refractive index response of the laser are investigated [60].

**Figure 19:**
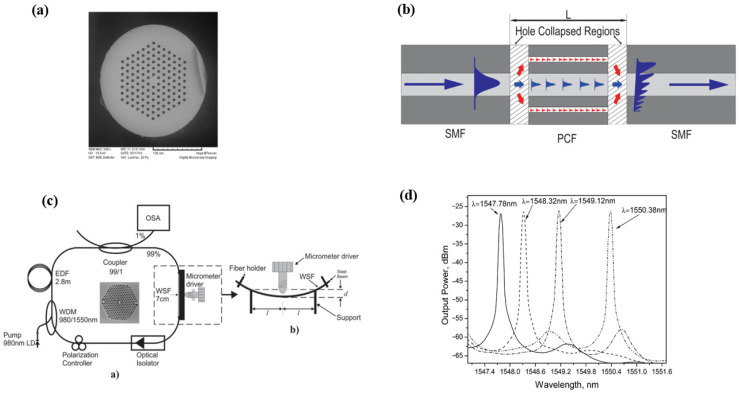
(**a**) The cross-section of the PCF; (**b**) Schematic diagram of the SMF-PCF-SMF filter [55]; (**c**) Experimental setup of the tunable fiber laser; (**d**) Tuning results of the single-wavelength output spectrum [63].

The similar MZI filter could be fabricated with photonic crystal fiber (PCF) [61,63]. Figure 19a,b show the SEM picture of the PCF and the schematic diagram of the SMF-PCF-SMF filter. As shown in Figure 19b, when the PCF and SMF were spliced together, the air hole of the PCF collapsed in the region of the splices. When the fundamental mode in the SMF was launched into the first collapsed region, cladding and core mode were excited. The cladding modes could be re-coupled to the core in the second collapse region, and interference occurred between the cladding and core modes. J. M. Sierra-Hernandez et al. [63] reported a tunable erbium-doped fiber laser based on this SMF-PCF-SMF filter. By changing the curvature radius of the MZI filter, the single-, double-, or triple-line emissions could be tuned from 1526 nm to 1550 nm. Figure 19c gives the experimental setup of the tunable fiber laser. Figure 19d shows the tuning results of the single-wavelength spectrum.

**Figure 20:**
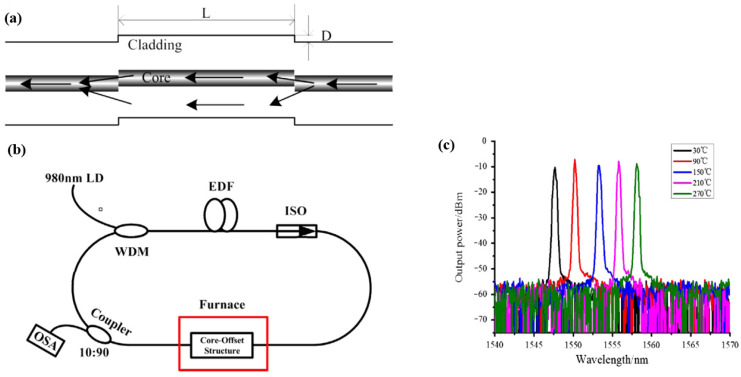
(**a**) Schematic diagram of the core-offset structure filter; (**b**) Experimental setup of the tunable fiber laser; (**c**) Spectral response against temperature [62].

When the light transmitted in the core of the SMF encountered a core-offset joint, part of the light would be coupled to the cladding, too [62,64,65,66]. X. Hao et al. [62] proposed a fiber laser temperature sensor based on SMF core-offset structure filter. The core-offset structure was fabricated by offset-splicing a section of SMF between two SMFs, as shown in Figure 20. When the light propagating in the SMF was launched into the first core-offset joint, part of the light was coupled to the cladding of the fiber. After a certain distance transmission, the light would be re-coupled to the core at the second core-offset joint. The filter was put on a furnace to change the temperature. When the temperature varied from 30 to 270, the central wavelength of the fiber laser changed from 1547.7 nm to 1558.1 nm. Y. Qi et al. [65] reported a wavelength-switchable fiber laser based on a few-mode fiber (FMF) filter with core-offset structure. FMF is a special multi-mode fiber with several core modes. The proposed fiber filter was fabricated by splicing a section of FMF between two SMFs. The light was injected into the FWF at the core-offset structure though the lead-in fiber. Different from SMF core-offset structure, several core modes were effectively excited in the FMF, which could be selected by adjusting the core-offset distance appropriately. And the excited cladding modes were lost by the absorption of the coating of FMF. Thus, interference occurs between different core modes at the second core-offset splicing region.

## 7. Applications of Multimode Interference Filters in Mode-Locked Fiber Lasers

**Figure 21:**
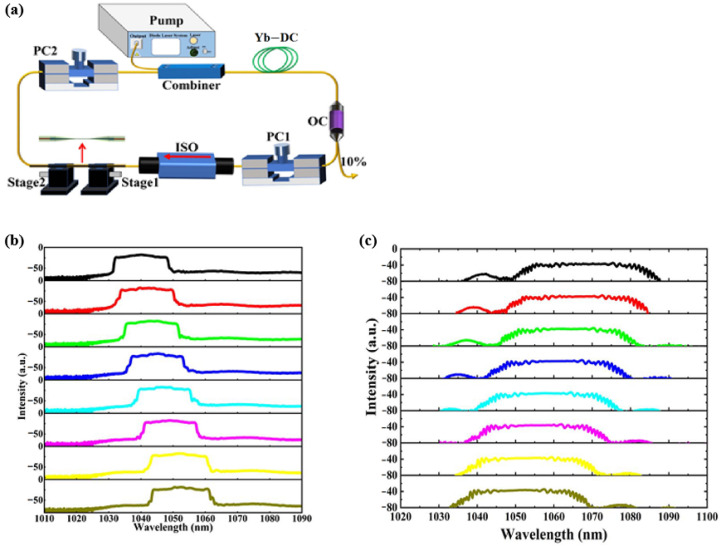
(**a**) Experimental setup diagram of the tunable mode-locked fiber laser based on tapered seven-core fiber filter; (**b**) Spectra of the tunable dissipative soliton fiber laser; (**c**) Spectra of the tunable amplifier similariton fiber laser [41].

In normal-dispersion mode-locked fiber lasers, the filter plays the role not only of wavelength selection but also of pulse reshaping. In 2006, A. Chong [67] reported a mode-locked ytterbium-doped fiber laser based on the spectral filtering of a highly chirped pulse in the cavity. By increasing the nonlinear phase shift accumulated by the pulse and inserting a spectral filter in the cavity, self-amplitude modulation via spectral filtering is enhanced. In another words, by cutting the edges of the spectrum of a chirped pulse, the filter helps reshape the pulse in the spectral domain. The filter and saturable absorber work together to maintain stable pulse operation. This type of pulse is generally in a Gaussian shape and is called dissipative soliton [68]. When the filter is narrower than 5 nm, another type of parabolic pulse may be obtained [69], which is a local nonlinear attractor in the gain segment of the oscillator and is usually referred to as parabolic amplifier similariton. Generally, both dissipative solitons and amplifier similaritons could be obtained in one mode-locked fiber laser. With the decrease in the filter bandwidth, the pulses will be switched from dissipative solitons to amplifier similaritons [70]. In 2023, our team reported a wavelength tunable fiber laser based on a TSCF filter. When a filter with a 3 dB bandwidth of 12.00 nm was selected, stable dissipative solitons were obtained. And when a filter with a 3 dB bandwidth of 6.64 nm was used, the fiber laser generated amplifier similaritons [41]. By applying tension to the tapered region, the central wavelengths of both dissipative solitons and self-similar pulses could be tuned. Figure 21a gives the experimental setup of the tunable fiber laser, and Figure 21b,c show the tuning results.

Another point worth to note is that the transmission spectrum of an interference filter is generally comb-shaped. The comb shape affects the spectrum of the pulse. When a narrow interference filter is adopted and the fiber laser delivers amplifier similaritons, the spectrum of the pulse usually exhibits modulated sidebands [7]. According to the experimental and simulation results, the sidebands in the modulated spectrum result from the comb-shaped oscillator transmission spectrum of the interference filter [7]. Therefore, when a multimode interference filter is used in a mode-locked fiber laser, the impact of filter bandwidth and shape on pulses should be considered.

As mentioned above, multi-mode fiber, no-core fiber, multi-core fiber, tapered fiber, and many other types of fibers have all been used to prepare multimode interference filters. Among them, filters based on multi-mode fibers and no-core fibers are less expensive and are more robust than tapered fiber filters. Multi-core fibers can be fused with traditional fibers using ordinary fusion splicers. However, the price of multi-core fiber is expensive. No special fiber is needed to fabricate fibers based on tapered fibers. Nevertheless, the tapering process requires special equipment.

## 8. Summary and Outlook

As a wavelength-selective device, the filter is one of the most important components in tunable fiber lasers. Compared with fiber grating and birefringent filters, multimode interference filters have the advantages of simple structure, flexible tuning methods, being easy to integrate with fiber devices, and flexible spectral ranges. The structures of multimode interference filters based on multimode fibers, no-core fibers, multi-core fibers, and tapered fibers have been introduced in this article, and the working principles of all these filters are analyzed. The research progress of tunable fiber lasers based on different structures of interference filters is summarized according to the classification of tuning methods.

With the advancement in fiber manufacturing and processing technology, the structure of multimode interference filters is continuously optimized, and the performance of tunable lasers is constantly improved. However, there is still a certain gap in the practical application of multimode interference filters. From a practical perspective, it is necessary to estimate their stability and immunity to the changing environments. In addition, the common methods to tune multimode fiber filters are stretching or bending, and the repeatability and reliability after multiple stretching or bending also need to be evaluated. Continuously improving the performance of multimode fiber interference filters and promoting the practicality of tunable fiber lasers is an important research direction.

In addition, mode-locked pulsed fiber lasers usually operate in the negative dispersion domain or positive dispersion domain. In a negative dispersion mode-locked laser, the filter determines the working wavelength of the laser, and the output wavelength is tunable by changing the peak transmission wavelength of the filter. In a positive dispersion mode-locked fiber laser, the peak transmission wavelength of the filter determines the working wavelength of the laser, and the bandwidth and shape of the filter also affect the evolution process and working mechanism of the pulse. The influence of the central transmission wavelength, bandwidth, and shape of the filter on the pulse formation process need to be studied to achieve the flexible output of the mode-locked laser with a tunable central wavelength and switchable pulse mechanism. This is another research direction for tunable fiber lasers.

## Figures and Tables

**Table 1:** Summary of the literature reports on tunable fiber lasers based on SMF-MMF-SMF filters.

Ref.	Structure	Tuning Method	Laser Type	Gain Fiber	Tuning Range
[10]	SMF-MMF-SMF	Stretching	Mode-locked	Yb-doped	NA
[13]	SMF-GIMF-SMF	Stretching	Mode-locked	Tm-doped	NA
[14]	Two cascaded SMF-MMF-SMF	Stretching	Optical parametric oscillator	Highly nonlinear fiber	1642.5–1655.4 nm
[16]	SMF-SIMF-GIMF-SMF	Bending	Mode-locked	Tm-doped	1835–1886 nm
[17]	SMF-SIMF-SMF	Bending	Mode-locked	Tm-doped	1834–1895 nm
[18]	SMF-MMF-SMF	Bending	Mode-locked	Er-doped	NA
[19]	SMF-MMF-SMF	Bending	Mode-locked	Tm-doped	1919.6–2014.9 nm
[20]	SMF-MMF-SMF	Bending	CW	Er-doped	1529–1581 nm
[21]	SMF-SIMF-SMF	Bending	Q-switched	Er-doped	1553–1569 nm
[22]	SMF-GIMF-SMF	Bending	Mode-locked	Yb-doped	1031.99–1039.32 nm
[23]	SMF-MMF-SMF	Wrapping into a PC	CW	Er-doped	1554.96–1564.25 nm;
[24]	Two cascaded SMF-MMF-SMF	Wrapping into a PC	CW	Er-doped	1533–1573 nm
[25]	SMF-GIMF-SMF	Wrapping into a PC	Mode-locked	Yb-doped	NA
[26]	SMF-MMF-SMF	Adjusting the PC in the cavity	CW	Tm-doped	1892–1916 nm
[27]	SMF-Taper GIMF-SMF	Adjusting the PC in the cavity	Mode-locked	Er-doped	NA
[28]	SMF-SIMF-GIMF-SMF	Adjusting the PC in the cavity	Mode-locked	Tm-doped	NA

NA: not available.

**Table 2:** Summary of the literature reports on tunable fiber lasers based on multi-core fiber filters.

Ref.	Structure	Tuning Method	Laser Type	Gain Fiber	Tuning Range
[33]	Two cascaded SMF-TCF-SMF	Bending and stretching	CW	Er-doped	1541.8–1560 nm
[34]	SMF-TCF-SMF	Bending	CW	Er-doped	1542.2–1566 nm
[35]	SMF-TSCF-SMF	Stretching	CW	Er-doped	1570.22–1559.33 nm
[36]	SMF-MMF-TCPCF-SMF	Stretching	CW	Er-doped	1549.32–1568.9 nm;
[38]	SMF-twin-core photonic crystal fiber (TCPCF)-SMF	Bending	CW	Er-doped	1560.4–1583.44 nm
[41]	SMF-TSCF-SMF	Stretching	Mode-locked	Yb-doped	Dissipative solitons: 1040.08–1052.44 nm, Amplifier similaritons: 1052.02–1068.16 nm

**Table 3:** Summary of the literature reports on tunable fiber lasers based on tapered fiber filters.

Ref.	Structure	Tuning Method	Laser Type	Gain Fiber	Tuning Range
[43]	Single cone	Stretching	Mode-locked	Tm-doped	1866.3–1916.4 nm
[44]	Single cone	Stretching	CW	Er-doped	1546–1566.5 nm
[45]	Single cone	Stretching	Mode-locked	Tm-doped	
[46]	Single cone	Stretching	CW	Er-doped	1910.6–1958.1 nm
[47]	Single cone	Stretching	Mode-locked	Er-doped	1560.6–1556.2 nm
[48]	Single cone	Adjusting the PC in the cavity	CW	Er-doped	NA
[49]	Single cone	Adjusting the PC in the cavity	Mode-locked	Yb-doped	NA
[50]	Double core	Bending	CW	Er-doped	1550–1605 nm
[51]	Double core	Bending	CW	Er-doped	1527–1563 nm
[52]	Double core	Bending	CW	Er-doped	
[53]	Double core	Bending	CW	Er-doped	Laser 1: 1528.778–1528.998 nm Laser 2: 1533.877–1534.096 nm
[54]	Double core	Bending	CW	Er-doped	NA
[55]	Double core	Changing the temperature of the glycerol solution surrounding the filter	CW	Er-doped	NA
[56]	Double core	Stretching	CW	Yb-doped	NA
[57]	Double core	Using a passive band pass filter	CW	Er-doped	1525–1562 nm
[58]	Double core	Adjusting the PC in the Sagnac loop	CW	Er-doped	1560.1–1612.8 nm

NA: not available.

## Data Availability

Data available upon request.
